# Status of Debtor Registration at an Enforcement Authority and Risk of Nonfatal Suicide Attempt

**DOI:** 10.1027/0227-5910/a000851

**Published:** 2022-02-09

**Authors:** Yerko Rojas

**Affiliations:** ^1^ School of Social Sciences, Södertörn University, Huddinge, Sweden

**Keywords:** suicidal behavior, debt repayment, default, bailiff, Sweden

## Abstract

**Abstract.** *Background:* Not much is known about whether paying unpaid debt is related to a reduced risk of suicidal behavior. *Aims:* To examine whether nonfatal suicide attempt varied by status of nonpayment of debt as registered at the Swedish Enforcement Authority (SEA). *Method:* People aged between 20 and 64 years with a registration date for an unpaid debt at the SEA during 2016 (*n* = 57,039) and registered as either active or inactive for a debt and/or a decision of debt reconstruction in the register in 2018 were followed up for a 2-year period for suicide attempt and compared with a sample from the general Swedish population (*n* = 301,714). *Results:* Those who were still active for a debt and/or a decision of debt reconstruction were about twice (*Odds Ratio* = 2.21) as likely to attempt suicide than those who no longer had an active debt in the SEA register. *Limitations:* The study was limited to suicide attempts that were registered as such in the National Patient Register. *Conclusion:* The results, based on unique nationwide register data, reinforce the importance of making tackling debt and financial distress part of current suicide prevention strategies. Professionals and others who interact with indebted people may be important gatekeepers in preventing suicide attempts.

There are growing calls to make *tackling debt and financial distress* a new strand in suicide prevention (Money and Mental Health Policy Institute, 2016). In fact, the detrimental impact of the last global financial crisis is still being felt by many people (Di Leo et al., 2018). Previous research has shown that financial hardship – in terms of unmanageable debt (Kameyama et al., 2011), debt repayment problems (Meltzer et al., 2011), insolvency (Kidger et al., 2011), and even evictions (Rojas & Stenberg, 2016) – is linked to suicidal behavior. However, whether getting out of a situation of not having been able to pay one’s debt is at all related to a lower risk of attempted suicide is not known (Richardson et al., 2013; Turunen & Hiilamo, 2014). This would seem to be an important knowledge gap, not least if one wants to meet the requirements needed to make tackling financial indebtedness part of the current selective suicide preventive strategies (World Health Organization [WHO], 2014).

With the help of the unique opportunity, offered by the Microdata Online Access (MONA) system at Statistics Sweden, to analyze nationwide register data, this study set out to examine whether the risk of nonfatal suicide attempt following a period of financial indebtedness between 2016 and 2017, among adults aged 20–64, varied with no longer being active for an unpaid debt and/or decision of debt reconstruction in the Swedish Enforcement Authority (SEA) register. To be registered for a debt-related issue at an enforcement authority is a reliable measure of financial indebtedness and is also thought to constitute a key stage of the indebtedness process, in terms of suicide ideation and attempted suicide (Bond & Holkar, 2018). The analysis adjusted for criminal, mental ill-health, demographic, and socioeconomic risk factors known to precede a registration at the SEA (Kronofogden, 2008; SOU, 2013). A comparison group comprising a matched sample of the Swedish population was also used.

The study assumed that paying unpaid debt registered at an enforcement authority is accompanied by a sense of restoration, control, and enhanced self-esteem, which implies a lower risk of suicide attempt compared to continuing in the vulnerable position of not being able to pay a debt registered at an enforcement authority. This assumption was based on recent sociohistorical assessments of the position of being unable to repay a debt – depicted as a very difficult and painful one for those who owed money, as their inability to restore themselves to parity (i.e., repay the debt) was viewed as an indication of personal inadequacy leading to feelings of self-blame (Graeber, 2014, p. 137). Having said that, and acknowledging that recovering from financial indebtedness takes time and may imply a long-term enhanced suicidogenic risk (Bond & Holkar, 2018), going from having been a debtor in default (i.e., not having paid a debt in time) to no longer having any active debt in the SEA register was also expected to be attached to an enhanced risk of suicide attempt compared to not having been registered at the SEA, at all, during the same study period.

## Method

### Cases: The SEA Register

The exposed group in this study included all adults aged 20–64 years, comprising both women and men, with a registration date for debt in the SEA register between January 1, 2016 and December 31, 2016. The SEA is a government agency with a range of responsibilities, including, but not limited to, debt collection, enforcement, and injunctions to pay (cf. Kronofogden, 2020). The SEA is responsible for both private and public (i.e., debts to central and local authorities) claims and registers all debts that have been confirmed through a simplified payment procedure or a court order and in respect of which enforcement has been attempted but has been unsuccessful (Jørgensen, 2016).

### A Matched Comparison Group: Other National Register Data

The data from the SEA register were linked to several other national registers. In this study, linkages made between the following registers were used: (a) the Medicinal Drug Register and the National Patient Register; (b) the register of persons convicted of criminal offences; (c) the longitudinal integration database for health insurance and labor market studies (known in Sweden as “LISA”); the population statistics register; and the geography database. These registers are administered by the National Board of Health and Welfare, the Swedish National Council for Crime Prevention and Statistics Sweden, respectively.

The comparison group was created by Statistics Sweden. With the aim of ensuring a nationwide comparison group, for each of the individuals registered at the SEA (i.e., for each of the individuals in the exposed group), a set of up to five controls was extracted (December 31, 2014) from the general Swedish population, matched by age, gender, and region of residence. The data set for the comparison group contained the same information from the national registers as the data set for the exposed group. The individuals registered at the SEA were removed from the sample population before the comparison group was created.

All the data were stored at Statistics Sweden and were made available to the author via its MONA system.

### Data Analysis

The first available information regarding whether a debtor has an active or inactive debt registered at the SEA is dated January 11, 2018. This means that the study allowed us to analyze the relationship between the status of a debt at an enforcement authority and suicide attempt for a follow-up period of approximately 2 years, as the available information on inpatient care for attempted suicide was limited to December 31, 2019. The use of 2-year follow-up periods to study attempted suicide after a given stage in the indebtedness process was in line with recent research (Kidger et al., 2011). The control variables were measured between 2014 and 2015, that is, during two calendar years preceding the registration date at the SEA, for both the exposed and the comparison group.

Personal identification numbers identified as erroneous were excluded, as well as those individuals who emigrated or died during the study period. The analysis only considered people for whom complete data were available on all the variables included in the models.

The relationship between the independent/control variables and attempted suicide was estimated using penalized maximum likelihood logistic regression (firthlogit). The loose-matching nature of the data under study allowed us to analyze them using unconditional logistic regressions (Kuo et al., 2018). The advantage with this form of analysis was the possibility of obtaining estimates for the matching variables (measured here on December 31, 2014), by simply including them as regular control variables in the analysis. Further, firthlogit is a method that is suitable for dealing with situations in which the event of interest is rare (cf. Rojas & Stenberg, 2016), which was the case here, in the sense that the proportion of attempted suicides in the data was lower than 5% (King & Zeng, 2001). Firthlogit is accessible as a sub-routine in STATA (Hilbe, 2009).

#### Dependent Variable

Attempted suicide was understood as being an external cause of morbidity, which, in accordance with the 10th revision of the International Classification of Diseases (ICD-10), is defined as recorded in the Swedish inpatient care register as either intentional self-harm (X60-X84) or an event of undetermined intent (Y10-Y34; cf. Almquist et al., 2020), at least once between January 12, 2018 and December 31, 2019.

#### Independent Variable

Examining all cases in which a nonpayment of debt, which has officially been registered, falls well within the administrative model of studying financial indebtedness (Turunen & Hiilamo, 2014), and is also what official governmental reports have described as the most reliable measure of over-indebtedness available in Sweden (SOU, 2013). In this study, *financial indebtedness* was divided into two subcategories:

1. *Registered at the SEA but no longer active in the register*: registered as not having an active debt in the SEA register on January 11, 2018, while appearing with a registration date for a debt in the SEA register for the year 2016; and
2. *Registered at the SEA and still active in the register*: registered as having an active debt and/or as having a decision of debt reconstruction at the SEA on January 11, 2018, and appearing with a registration date for a debt in the SEA register for the year 2016.

#### Control Variables

The control variables were measured in accordance with the analytical strategy described above and were defined as follows. *Gender:* women/men; *age:* year of birth; *region of residence*: living in one of the three regions in Sweden that includes the country’s three largest cities (Stockholm, Gothenburg, and Malmö, respectively); *place of birth:* foreign born/born in Sweden; *family status*: single persons/all types of family constellations (married families [including civil unions], cohabiting families, and one-parent families); *education*: pre-upper-secondary, upper-secondary, and post-upper-secondary education; *unemployment:* being registered as unemployed at the relevant authorities for at least 1 day over the course of a 2-year period; *social welfare recipiency:* having received means-tested social assistance at least once over the course of 2 years; and finally, *mental illness,* which was measured using two different indicators – *antidepressants* (registered as having been prescribed antidepressants [ATC-code: N06A] at least once over a 2-year period), and *inpatient care* (having been recorded in the Swedish inpatient care register with mental, behavioral, or neurodevelopmental disorders as a main diagnosis [ICD-10: F01-F99] at least once over a 2-year period).

## Results

The study base was composed of an exposed group (that is, people with a registration date for a debt at the SEA during 2016 and registered in the SEA register in 2018 as either active or inactive for debt and/or a decision of debt reconstruction) and a matched comparison group from the Swedish population, comprising a total of 16,920, 40,119, and 301,714 individuals, respectively (see Table 1). A total of 557 nonfatal suicide attempts were included in the analysis. Of these, 161 occurred among those registered as having an active debt and/or as having a decision of debt reconstruction at the SEA on January 11, 2018. Additionally, 96 attempts occurred among those registered as not active at the SEA on January 11, 2018, and 300 among those who did not appear at all in the SEA register on January 11, 2018 or did not have a registration date for a debt at the SEA in 2016 (see Table 1).

**Table 1:** Distribution of dependent and control variables included in the models by the group that had a registration date for a debt at the Swedish Enforcement Authority (SEA) during 2016 (exposed group) and the matched sample of the Swedish population (comparison group)

Variable	Respondents		
	Exposed group		Comparison group	
	Registered for a debt at SEA in 2016 and still active in the register in 2018 (*n* = 16,920)	Registered for a debt at SEA in 2016 and no longer active in the register in 2018 (*n* = 40,119)	Not registered for a debt at SEA in 2016, nor found in the register in 2018 (*n* = 301,714)
Dependent variable – measured in 2018 and 2019			
Nonfatal suicide attempt (%)	0.95	0.24	0.10
Control variables – measured in 2014			
Place of birth (%)			
Born in Sweden	65.83	74.29	81.96
Born abroad	34.17	25.71	18.04
Age (*M*)			
Age in January 2016	36.74	38.73	38.33
Gender (%)			
Men	57.97	61.51	60.86
Women	42.03	38.49	39.14
Region of residence (%)			
Living in a big city	54.34	56.71	56.44
Control variables – measured in 2015			
Education (%)			
Post-upper secondary education	15.84	32.71	41.59
Upper secondary	54.56	50.88	48.26
Pre-upper secondary	29.60	16.41	10.15
Single-person household (%)			
Single	27.42	21.08	18.01
Control variables – measured in 2014 and 2015			
Unemployment (%)			
Unemployed	37.60	22.91	15.15
Social welfare recipiency (%)			
Received social assistance	22.45	10.31	4.41
Criminality (%)			
Convicted of a criminal offence	7.58	4.99	1.24
Mental illness (%)			
Prescribed antidepressants	23.99	15.78	9.89
Inpatient care (mental/behavioral/neurodevelopmental disorders)	5.85	2.32	0.77

The proportion of people who attempted suicide in the still-active group was approximately 4 times as large as the corresponding proportion in the group that was no longer active in the SEA and about 10 times as large as the corresponding proportion in the group that did not appear at all in the SEA (see Table 1). Apart from the matching variables of age, gender, and region of residence, the distributions of the control variables were clearly different between the exposed group and the comparison group, confirming the adverse conditions of those people who experienced financial indebtedness (see Table 1).

The results from the penalized maximum likelihood logistic regression analysis are presented in Table 2. In Model 1, we can see that the risk of suicide attempt differs, in a statistically significant way, by status of indebtedness. People who had a registration date for a debt at the SEA at any point during 2016 and were still active (for a debt and/or a decision of debt reconstruction) in the register on January 11, 2018 were approximately four times more likely to attempt suicide than people who had a registration date for a debt at the SEA in 2016 but were no longer registered as active in the register on January 11, 2018 (*OR* = 4.00; 95% CI [3.10, 5.15]). People who did not have a registration date for a debt at the SEA at all in 2016, nor found in the authority’s register on January 11, 2018, on the other hand, had about 59% lower odds of attempting suicide than those who, although no longer active in the register on January 11, 2018, had a registration date for a debt at the SEA in 2016 (*OR* = 0.41; 95% CI [0.33, 0.52]).

**Table 2:** Logistic regression of financial indebtedness and nonfatal suicide attempt among adults aged 20–64 in Sweden 2018–2019

Variable	Model 1 Crude *OR* [95% CI]	Model 2 Adjusted *OR* [95% CI]	Model 3 Adjusted *OR* [95% CI]	Model 4 Adjusted *OR* [95% CI]	Model 5 Adjusted *OR* [95% CI]
Independent variable					
Financial indebtedness					
Registered for a debt at the Swedish Enforcement Authority (SEA) in 2016 and still active in the register in 2018 Not registered for a debt at SEA in 2016, nor found in the register in 2018 Ref: registered for a debt at SEA in 2016 and no longer active in the register in 2018)	4.00 [3.10, 5.15]* 0.41 [0.33, 0.52]*	3.65 [2.83, 4.70]* 0.41 [0.32, 0.51]*	2.81 [2.17, 3.64]* 0.50 [0.39, 0.63]*	2.21 [1.70, 2.87]* 0.63 [0.50, 0.80]*	2.55 [1.88, 3.46]* 0.48 [0.37, 0.63]*
Control variables					
Age					
Year of birth (continuous)		1.03 [1.03, 1.03]*	1.02 [1.02, 1.03]*	1.02 [1.02, 1.03]*	1.02 [1.02, 1.03]*
Gender					
Men (ref: women)		0.57 [0.48, 0.68]*	0.53 [0.45, 0.63]*	0.69 [0.58, 0.82]*	0.69 [0.58, 0.82]*
Place of birth					
Born in Sweden (ref: foreign born)		1.49 [1.18, 1.88]*	1.88 [1.48, 2.40]*	1.33 [1.04, 1.69]*	1.38 [1.09, 1.76]*
Region of residence					
Living in a big city (ref: other)		0.77 [0.65, 0.91]*	0.83 [0.70, 0.98]*	0.80 [0.67, 0.94]*	0.80 [0.68, 0.95]*
Single-person household					
Single (ref: other family constellations)		2.00 [1.68, 2.39]*	1.96 [1.64, 2.34]*	1.51 [1.25, 1.81]*	1.48 [1.23, 1.77]*
Unemployment					
Unemployed (ref: other)			1.13 [0.92, 1.39]	1.12 [0.92, 1.37]	1.11 [0.91, 1.35]
Social welfare recipiency					
Received social assistance (ref: other)			1.91 [1.50, 2.44]*	1.25 [0.98, 1.59]	1.30 [1.02, 1.65]*
Education					
Post-upper secondary education (ref: pre-upper secondary)			0.39 [0.30, 0.50]*	0.45 [0.34, 0.58]*	0.47 [0.36, 0.61]*
Upper secondary (ref: pre-upper secondary)			0.60 [0.50, 0.74]*	0.66 [0.54, 0.81]*	0.67 [0.55, 0.83]*
Criminality					
Convicted of a criminal offence (ref: other)			1.94 [1.40, 2.68]*	1.10 [0.78, 1.54]	1.20 [0.86, 1.67]
Mental illness					
Prescribed antidepressants (ref: other)				3.73 [3.07, 4.53]*	3.50 [2.88, 4.26]*
Inpatient care (mental/behavioral/neurodevelopmental disorders) (ref: other)				8.16 [6.51, 10.23]*	5.31 [3.29, 8.57]*
Interaction term: Inpatient care × still active SEA Inpatient care × not registered in SEA					0.80 [0.45, 1.42] 3.19 [1.87, 5.45]*
Nonfatal suicide attempts	557	557	557	557	557
Total study population (*n*)	*n* = 358,753	*n* = 358,753	*n* = 358,753	*n* = 358,753	*n* = 358,753
*Note*. Ref = reference group. *Statistically significant (at the 5% level).					

As can be seen in Model 2, Model 3, and Model 4, this relationship remained statistically significant when adjusted for age (including a quadratic term for age that was not significant and hence not included in the final model), gender, place of birth, region of residence and single-person household, unemployment, social welfare recipiency, education, criminality, prescription of antidepressant, and inpatient care due to mental illness. However, when checking for possible interactions between financial indebtedness and the control variables that remained statistically significant in Model 4, the effect of status of financial indebtedness was found to vary, in part, with inpatient care due to mental illness at least once during the two calendar years preceding the date of registration at the SEA. The results of the interaction analysis are presented in Model 5, which was also the final model and to be considered as the multiplicative counterpart of Model 4, that is, the same model plus relevant interaction term.

Model 5 shows that the difference found in the odds of nonfatal suicide attempt between those who were still active in the register on January 11, 2018, and those who were no longer registered as active but also had a registration date for a debt at the SEA in 2016 did not vary with earlier inpatient care due to mental illness (see the nonsignificant interaction term in Model 5). Hence, the estimated odds ratio for this comparison presented in Model 4 (*OR* = 2.21; 95% CI [1.70, 2.87]) could be considered a valid final approximation of the relation in question, that is, people who were still active for a debt and/or a decision of debt reconstruction at the SEA were more than twice as likely to attempt suicide than people who were no longer registered as active in the register. By contrast, the final odds of attempting suicide for people who did not have a registration date for a debt at the SEA at all in 2016, nor found in the authority’s register on January 11, 2018, compared to those who, although no longer active in the register, had a registration date for a debt at the SEA in 2016, were still about 50% lower but only statistically significant among people who did not receive inpatient care prior to the date of registration at the SEA (*OR* = 0.48; 95% CI [0.37, 0.63], Model 5).

## Discussion

This study was the first to examine the relationship between debt repayment and nonfatal suicide attempt, using large-scale register data for an entire country (Bond & Holkar, 2018; Richardson et al., 2013; Turunen & Hiilamo, 2014). This made it possible to control, in an unprecedented way, for crucial factors that may have been confounding the relationship, including mental illness prior to the date of registration at an enforcement authority (such as depression and inpatient care). The fact that going from being a debtor in default to no longer being active in the SEA register seemed, on the one hand, to imply a lower risk of suicide attempt, compared to continuing to have an active debt and/or a decision of debt reconstruction at the SEA, adjusting for multiple background variables. On the other hand, it implies an enhanced risk of suicide attempt, compared to not appearing at all in the SEA register – at least for those who did not receive inpatient care prior to the date of registration at the SEA – has strong policy implications, two of which are particularly important.

First, we need to acknowledge financial indebtedness as a risk factor, in its own right, for suicidal behavior. Both the efforts toward preventing financial indebtedness from occurring altogether and the efforts directed toward helping people to recuperate from debt repayment failure may be important to break the link between financial indebtedness and suicide attempt (Bond & Holkar, 2018). In fact, debt counselling and other programs for resolving debt-related problems that are currently being put forward as necessary for alleviating the adverse effects of indebtedness on health, in general (Turunen & Hiilamo, 2014), might also apply to suicidal behavior.

Second, professionals and others who interact with individuals who have debt problems may be important gatekeepers in preventing suicide attempt. A gatekeeper is anyone who is in a position to identify whether a person may be contemplating suicide (WHO, 2014). Gatekeepers are of particular importance when individuals at risk of dying by suicide do not seek help but may exhibit risk factors and behaviors that identify them as being vulnerable when encountered (WHO, 2014). This has been suggested to be the case for people experiencing unmanageable debt, as the shame they feel for their situation coupled with the demand for debt repayment prevent/distract them from focusing on their mental well-being and engaging in help-seeking behavior (Kameyama et al., 2011). In Sweden, municipalities are expected to provide budget and debt advice to people who are indebted – both to prevent over-indebtedness and to help them find a solution to their problems. Thus, developing the knowledge, attitudes, and skills of social service workers in identifying individuals at risk of suicide (e.g., individuals with undiagnosed/untreated depression), determining the level of risk, and then referring at-risk individuals for treatment might be particularly important (Bond & Holkar, 2018; WHO, 2014).

### Limitations

Two main methodological considerations should be borne in mind when interpreting the results of this study. First, the study was limited to suicide attempts that were registered as such in the Swedish National Patient Register. In practice, this restricted the extent to which the results could be extrapolated beyond the confines of these specific types of registered acts. A great many of the suicide attempts do not come to the attention of health authorities (WHO, 2002). In fact, it has been estimated that only half of all suicide attempts in Sweden lead to hospital care (Rojas, 2013).

Second, because the study was entirely based on register data, there was a lack of self-reported information on possible confounders that might have influenced the results (Thygesen & Ersbøll, 2014), for example, attitudes toward debt, money-management practices, and the like. However, capturing representative and sizeable groups of individuals with this type of debt problem through traditional surveys and follow-ups, in terms of suicide attempts, has thus far proven to be very difficult (Richardson et al., 2013; Turunen & Hiilamo, 2014; Webley & Nyhus, 2001), which makes register-based studies of the kind presented here all the more important (Thygesen & Ersbøll, 2014).

## Conclusion

Financial indebtedness has a detrimental impact on individuals’ risk of nonfatal suicide attempt. However, going from being a debtor in default to no longer being active in the SEA register seems to imply a lower risk of suicide attempt, compared with continuing to have an active debt and/or a decision of debt reconstruction at the enforcement authority – adjusting for well-known suicidogenic risk factors present prior to the date of registration at the enforcement authority’s register. Although previous studies have recognized the connection between financial indebtedness and suicidal behavior, this was the first to examine the relationship between repayment of unpaid debt and suicide attempt, using large-scale register data for an entire country.

## Notes

**Publication Ethics:** The research is approved by the Swedish Ethical Review Authority (Reference Number: dnr 2017/2227–31/5).
